# SADI-S as a Conversion for Suboptimal Clinical Response and Recurrent Weight Gain Following Bariatric Surgery: A Retrospective Cohort Study

**DOI:** 10.1007/s11695-025-08483-3

**Published:** 2026-01-26

**Authors:** Reut El-On, Adi Litmanovich, Adam Abu-Abeid, Idan Carmeli, Shlomi Rayman, Shai Meron Eldar, Andrei Keidar

**Affiliations:** 1 Division of General Surgery, Tel Aviv Sourasky Medical Center, Tel Aviv, Israel; 2 The faculty of Medical and Health Science, Tel-Aviv University, Tel Aviv, Israel; 3 Department of General Surgery, Assuta Ashdod Public Hospital, Ashdod, Israel; 4 Department of Surgery, Laniado Hospital, Sanz Medical Center, affiliated with the Adelson School of Medicine, Ariel University, Netanya, Israel

**Keywords:** SADI-S, Bariatric surgery, Recurrent weight gain, Suboptimal clinical response, Conversion surgery

## Abstract

**Background:**

Recurrent weight gain (RWG) and Suboptimal Clinical Response (SCR) after primary metabolic and bariatric surgery (MBS) are common, often necessitating revisional procedures. Single-anastomosis duodeno-ileal bypass with sleeve gastrectomy (SADI-S) has emerged as a promising conversion option.

**Methods:**

We retrospectively reviewed patients who underwent laparoscopic conversion to SADI-S for RWG/SCR between 2018 and 2024. Eligible patients had prior MBS after meeting national guidelines. Data included demographics, weight-loss outcomes, complications and nutritional/metabolic markers. The primary outcomes were percent of total weight loss (%TWL) and excess weight loss (%EWL). Long-term complications and need for revisional surgery were also assessed.

**Results:**

Sixty-nine patients (mean age 42.7 ± 9.8 years; 66.7% female) were included. Most (65.2%) were converted from sleeve gastrectomy. Mean follow-up was 2.2 ± 1.4 years. Mean %TWL was 36.4%, 37.8%, and 34.5% at 1, 3, and 5 years, respectively, and %EWL exceeded 90% at all time points. Major complications (Clavien-Dindo ≥ 3b) occurred in 5.8% within 90 days, with 4 reoperations in the perioperative period. No conversional surgeries were required during the follow-up period.

**Conclusions:**

Conversion to SADI-S is a safe and effective option for RWG/SCR following primary MBS, yielding durable weight loss and metabolic improvement with low complication rates. These findings support its use as a conversion strategy, warranting further prospective validation.

**Supplementary Information:**

The online version contains supplementary material available at 10.1007/s11695-025-08483-3.

## Key Points

Conversion to SADI-S achieved durable weight loss with %TWL > 30% up to 5 years.

Major complication rates were low.

Conversion to SADI-S was shown to be safe in terms of nutritional status.

Outcomes were consistent regardless of the type of prior bariatric procedure.

**Supplementary Information:**

The online version contains supplementary material available at 10.1007/s11695-025-08483-3.

## Introduction

Recurrent weight gain (RWG) and Suboptimal Clinical Response (SCR) remain persistent issues following primary metabolic and bariatric surgery (MBS). Reported rates vary widely depending on the definition and index procedure, with studies indicating that approximately 20–40% of patients experience clinically significant weight regain or insufficient weight loss within 2–6 years postoperatively [1–6]. Consequently, conversion procedures are increasingly common, ranging from 4.5% after Roux-en-Y gastric bypass (RYGB) to as high as 75% after restrictive procedures like sleeve gastrectomy (SG) and laparoscopic adjustable gastric banding (LAGB) [7]– [8].

Single-anastomosis duodeno-ileal bypass with sleeve gastrectomy (SADI-S) has gained prominence both as a primary and revisional MBS technique, including for weight-related problems, such as RWG/SCR [9–15]. Although conversion procedures generally yield less favorable outcomes than primary MBS [16], evidence specifically evaluating conversion to SADI-S remains limited. While SADI-S has shown excellent results as a primary procedure, data on its effectiveness and safety as a revisional option - particularly concerning metabolic and nutritional outcomes - are scarce.

This study aims to assess the outcomes of SADI-S as a conversion procedure for RWG/SCR, focusing on weight-loss efficacy and long-term complications. We hypothesize that conversion to SADI-S offers a safe and durable solution with satisfactory outcomes.

## Methods

Following Institutional Review Board (IRB) approval, a retrospective review was performed on a prospectively maintained database including all patients who underwent conversion to SADI-S for RWG/SCR between January 2018 and December 2024 at a single tertiary center. Inclusion criteria encompassed adult patients with a history of previous MBS and documented RWG (defined as > 25% regain of lost weight from nadir) or SCR (defined as percent of total weight loss (TWL) < 20% or percent of excess weight loss (EWL) < 50%), and patients who qualified for MBS conversion according to the 2022 ASMBS and IFSO criteria [17]. Excluded were those converted to other procedures or those converted for non-weight-related indications such as GERD, strictures or anastomotic dysfunction.

Data collected included demographics, anthropometrics, obesity-related conditions, prior MBS history, intra-operative and post-conversion complications, and bowel movement frequency. Nutritional/metabolic markers included HbA1c, hemoglobin, albumin, iron, ferritin, transferrin, folate, vitamin B12, vitamin D, lipid profile, and the need for iron transfusions. Symptom burden and medication/supplement use were assessed via clinic records. All patients underwent multidisciplinary preoperative assessment and optimization by surgeons, dieticians, anesthesiologists and social workers. Upper gastrointestinal (UGI) endoscopy or other UGI studies were routinely performed.

Postoperative complications were categorized using the Clavien-Dindo system, with ≥ Grade 3b considered major. Long-term nutritional and metabolic complications were defined as any clinical symptoms or laboratory findings indicative of deficiencies. Weight loss outcomes were assessed using %TWL and %EWL, calculated from the pre-conversion weight to the last follow-up. A %TWL greater than 20% was considered satisfactory.

### Surgical Technique

All conversions were performed laparoscopically using standard port placements. The SADI-S technique followed the method described by Sánchez-Pernaute [18], with modifications based on intraoperative findings, since all SADI-S procedures in this study were performed as conversion surgeries, requiring specific and individual adjustments as following.

## SADI-S Technique

The greater omentum was mobilized beginning approximately 4 cm distal to the pyloric sphincter and continuing toward the left crus of the diaphragm. A 36-French bougie was then inserted along the greater curvature of the stomach to calibrate the sleeve. Gastric transection was performed 4 cm proximal to the pylorus. Subsequently, the first portion of the duodenum was transected approximately 4 cm distal to the pyloric sphincter, and a preparatory stay suture was placed at the proximal transected end of the duodenum to facilitate later anastomosis.

The cecum and terminal ileum were identified, and the small bowel length was measured proximally from the terminal ileum for a distance of 250 cm, defining the length of the common channel. The total small bowel length was not routinely measured or documented intraoperatively. An end-to-side, hand-sewn, double-layered duodeno-ileal anastomosis was then constructed between the proximal duodenum and the omega loop of the ileum, using continuous absorbable sutures to establish continuity for the passage of biliopancreatic secretions and ingested food.

A routine blue-dye leak and patency test was performed for both the gastric sleeve and the duodeno-ileal anastomosis to ensure integrity and absence of obstruction. Mesenteric defects were not closed on a routine basis.

## Specific Considerations

### Sleeve Resizing

**Fig. 1:**
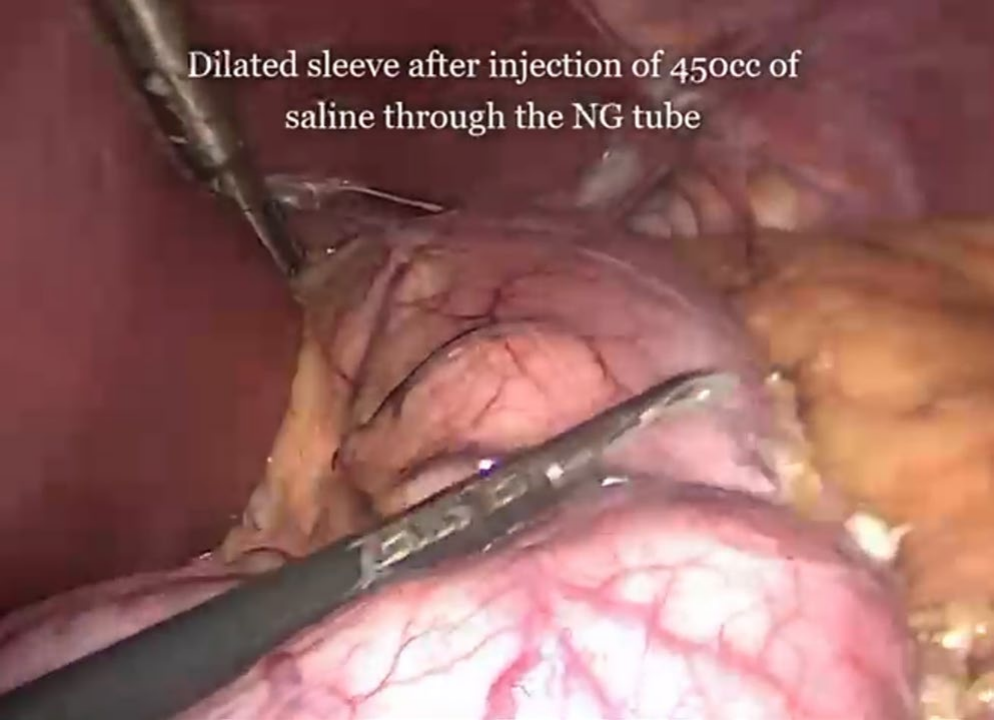
Intraoperative evaluation of sleeve dilation. Intraoperative view of a dilated gastric sleeve after injection of 450 mL saline through the nasogastric tube

A sleeve resizing procedure was undertaken in cases where suggestive anamnesis (the ability to consume a 200 g steak), preoperative imaging (Upper GI series, CT volumetry) or intraoperative evaluation (filling the sleeve with saline) demonstrated gastric dilation (Fig. 1). During surgery, the gastric sleeve was completely mobilized up to the left crus of the diaphragm, allowing thorough inspection of the hiatus for the presence of a diaphragmatic hernia. A 36-French bougie was then introduced along the lesser curvature to calibrate the gastric lumen, and gastric transection was subsequently performed using a linear stapler guided precisely against the bougie.

### Conversion from OAGB/RYGB to SADI-S[19]

The gastrojejunal anastomosis was transected using a linear stapler, after which the gastric remnant was resected and a hand-sewn gastro-gastrostomy was constructed. Subsequently, the duodeno-ileal anastomosis was performed as aforementioned. In all conversions from OAGB/RYGB, ICG was used in effort to preserve the right gastric artery.

### Band Removal

Conversion from LAGB was preceded by removal of the band and fibrous capsule, unless previously performed.

## Statistical Analysis

Categorical variables were summarized as events and percentages. Continuous variables were presented as means ± standard deviation. Comparisons of pre- and post-conversion data were performed using Chi-squared or Fisher’s exact tests for categorical variables, and paired t-tests or Mann-Whitney U tests for continuous variables, based on distribution. Statistical significance was defined as *p* < 0.05.

## Results

A total of 69 patients underwent conversion to SADI-S for RWG/SCR within the study period. Baseline characteristics and are shown in Table 1. The mean age was 42.7 ± 9.8 years, and 66.7% were female. Most patients had undergone a single prior MBS (85.5%), with a mean interval of 9.2 ± 3.4 years between prior MBS and conversion to SADI-S. Sleeve gastrectomy was the most common primary procedure (65.2%), followed by laparoscopic adjustable gastric banding (LAGB) (18.8%), Roux en Y Gastric Bypass (RYGB) (11.6%), and One Anastomosis Gastric Bypass (OAGB) (4.3%).

**Table 1:** Baseline characteristics

Total number of patients	*n* = 69
Characteristic	
Age at conversion, mean ± SD	42.7 ± 9.8
Female gender, n (%)	46 (66.7%)
Weight Before 1 st MBS, mean ± SD	129 ± 23.1
BMI Before 1 st MBS, mean ± SD	46.8 ± 6.8
BMI > 50, n (%)	25 (36.2%)
Weight Before SADI-S, mean ± SD	119.8 ± 22.7
BMI Before SADI-S, mean ± SD	43.1 ± 6.5
BMI > 50, n (%)	12 (17.4%)
Past MBS, n (%)	
1	59 (85.5%)
2	10 (14.5%)
Type of past MBS, n (%)	
LAGB	13 (18.8%)
LSG	45 (65.2%)
with Re-Sleeving	38/45 (84.4%)
w/o Re-Sleeving	7/45 (15.6%)
OAGB	3 (4.3%)
RYGB	8 (11.6%)
Interval from past MBS to SADI-S (years), mean ± SD	9.2 ± 3.4
Obesity-related diseases, n (%)	
T2D*	5 (8.3%**)
Hypertension	9 (13%)
Fatty liver	49 (71%)
OSA	8 (11.6%)
Dyslipidemia	30 (46.9%**)
Reflux	26 (39.4%**)

* HbA1C ≥ 6.5 according to ADA and WHO

** Pre-conversion HbA1C was available in 60 patients, dyslipidemia in 64 patients, reflux in 66 patients

*MBS* metabolic and bariatric surgery, *BMI* body mass index, *SADI-s* Single-anastomosis duodeno-ileal bypass with sleeve gastrectomy, *LAGB* Laparoscopic Adjustable Gastric Banding, *OAGB* One Anastomosis Gastric Bypass, *RYGB* Roux en Y Gastric Bypass, *T2D* type 2 diabetes, *OSA* Obstructive Sleep Apnea

As noted, sleeve gastrectomy was the most common prior bariatric procedure preceding SADI, accounting for 45 patients (65.2%). Among these patients, re-sleeving (trimming of a pre-existing sleeve) was performed in 38 cases (84.4%) (Table 1). The decision-making algorithm guiding re-sleeve performance is detailed in the Methods section.

The mean BMI prior to primary MBS was 46.8 ± 6.8 kg/m², and the mean BMI prior to conversion was 43.1 ± 6.5 kg/m². The mean maximal weight loss after previous surgery (at nadir, before starting weight gain) was 42.1 kg, and the mean BMI units loss was 15.2. Common obesity-related diseases included type 2 diabetes (8.3%), hypertension (13%), fatty liver (71%), obstructive sleep apnea (11.6%) and dyslipidemia (46.9%). Reflux treated with proton pump inhibitors was prevalent in 39.4% of patients.

The perioperative outcomes of patients are shown in Table 2. Of the 69 patients, no intraoperative complications or perioperative mortality occurred. Within 90 days postoperatively, complications were reported in 8 patients (11.6%), including leaks (7.2%), obstruction/stricture (1.4%), stenosis with incarcerated hiatal hernia (1.4%), and ulcer (1.4%). According to the Clavien-Dindo classification, 4 patients (5.8%) experienced complications classified as ≥ 3b, requiring early revisional surgery; 3 leaks (from the re-sleeve staple line) were treated by operative drainage and 1 case of stenosis with incarcerated hiatal hernia was treated surgically as well. One suspected leak originating from the duodeno-ileal (DI) anastomosis was identified radiologically, without associated clinical manifestations, and was successfully managed conservatively with no subsequent sequelae. No ICU admissions were required, and no clinically evident DI leaks were observed. Another radiologically suspected, clinically silent leak of unclear origin was managed conservatively. Notably, all Clavien–Dindo complications classified as ≥ 3b occurred in patients who underwent re-sleeving, underscoring the procedural significance of this step.

**Table 2:** Perioperative outcomes

Total number of patients	*n* = 69
Characteristic	
Intraoperative complications, n (%)	0 (0%)
90 days complications, n (%)	8 (11.6%)
Leak	5/8 (7.2%)
Obstruction/Stricture	1/8 (1.4%)
Stenosis + incarcerated hiatal hernia	1/8 (1.4%)
Ulcer	1/8 (1.4%)
CD group	
CD < 3b, n (%)	4 (5.8%)
CD ≥ 3b, n (%)	4 (5.8%)
ICU admission, n (%)	0 (0%)
Revisional surgery, n (%)	4 (5.8%)
Perioperative mortality, n (%)	0 (0%)

*CD* Clavien-Dindo

**Fig. 2:**
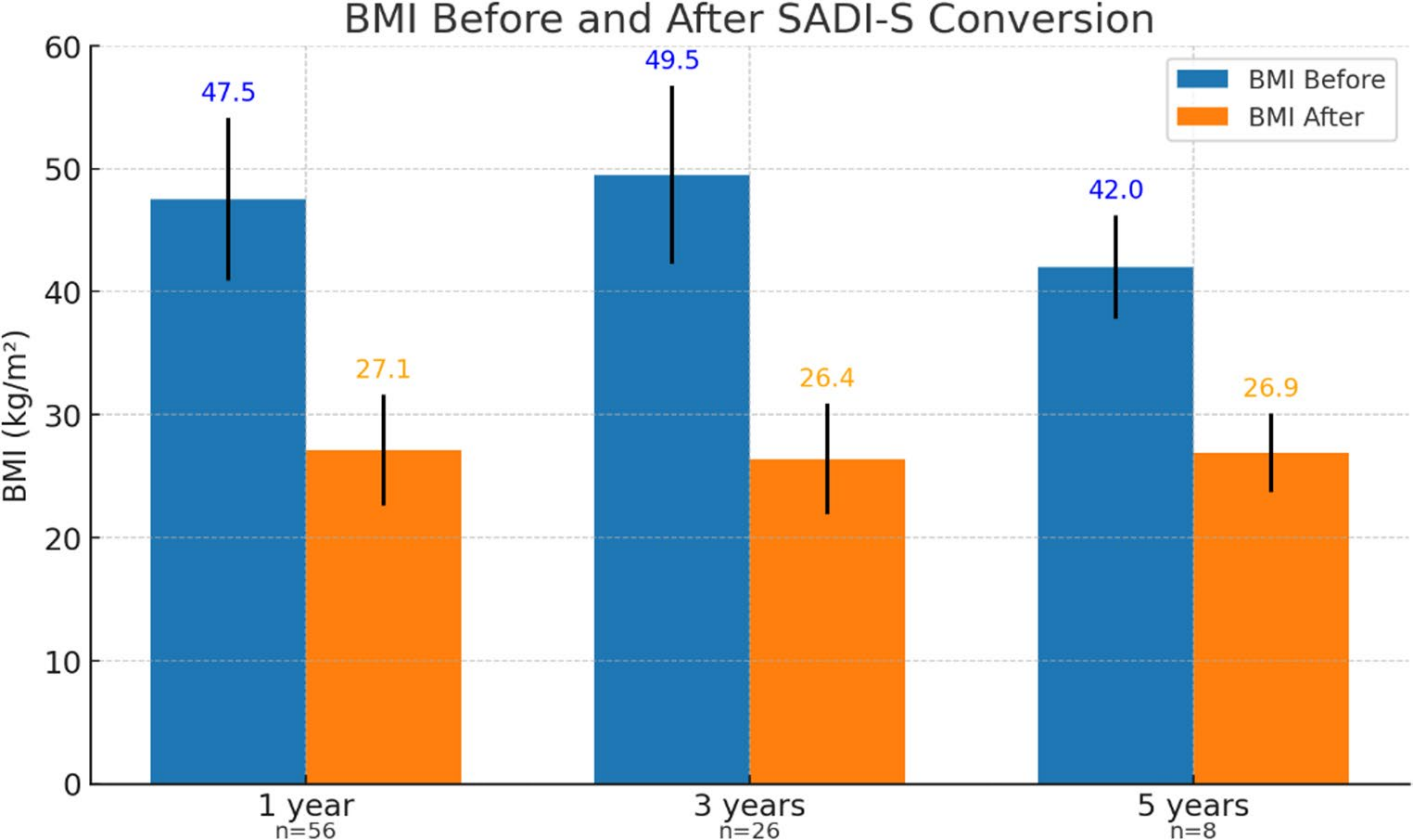
BMI before and after conversion to SADI-S at 1, 3, and 5 years of follow-up. Mean BMI values (±SD) are shown for each timepoint before and after conversion surgery. BMI decreased significantly from baseline at all follow-up points: from 47.5 kg/m² to 27.1 at 1 year (n=56), 49.5 to 26.4 at 3 years (n=26), and 42.0 to 26.9 at 5 years (n=8). Error bars represent standard deviation. *BMI*, body mass index; *SADI-S*, Single-anastomosis duodeno-ileal bypass with sleeve gastrectomy

**Fig. 3:**
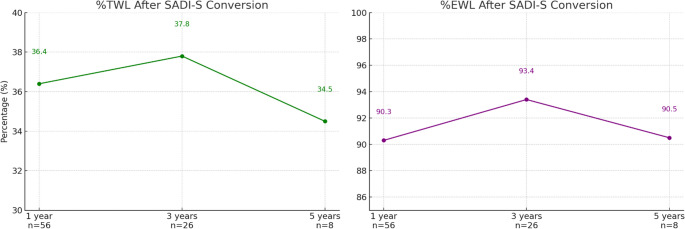
Total and excess weight loss after SADI-S conversion at 1, 3, and 5 years of follow-up*. *Left panel shows mean percent total weight loss (%TWL), which remained above 34% at all time points: 36.4% at 1 year (n=56), 37.8% at 3 years (n=26), and 34.5% at 5 years (n=8). Right panel shows mean percent excess weight loss (%EWL), consistently exceeding 90%: 90.3% at 1 year, 93.4% at 3 years, and 90.5% at 5 years. Data reflect maintained weight loss over time following conversion to SADI-S.*SADI-S*, Single-anastomosis duodeno-ileal bypass with sleeve gastrectomy

The long-term metabolic and surgical outcomes of patients are depicted in Table 3. At a mean follow-up of 2.2 ± 1.4 years, data were available for 56 patients at 1 year (81.2%), 26 patients at 3 years (37.7%), and 8 patients at 5 years (11.6%). Mean BMI decreased from 47.5 ± 6.6 to 27.1 ± 4.5 kg/m² at 1 year, from 49.5 ± 7.2 to 26.4 ± 4.5 at 3 years, and from 42.0 ± 4.2 to 26.9 ± 3.2 at 5 years (Fig. 2). Corresponding mean %TWL was 36.4% ± 10.8%, 37.8% ± 12% and 34.5% ± 5.9% at 1,3 and 5 years, respectively, and %EWL was 90.3% ± 24.1%, 93.4% ± 23.7% and 90.5% ±18.4% at 1,3 and 5 years, respectively (Fig. 3). One patient at 1 year (1.8%) and 1 patient at 3 years (3.8%) had %TWL < 20%. All 8 patients at 5-years F/U achieved %TWL > 20%.

**Table 3:** Long-term follow-up (mean, SD − 2.2 ± 1.4 years)

Characteristic	1 year	3 years	5 years
	*n* = 56 (81.2%)	*n* = 26 (37.7%)	*n* = 8 (11.6%)
Metabolic			
BMI before conversion, mean ± SD	47.5 ± 6.6	49.5 ± 7.2	42.0 ± 4.2
BMI after conversion, mean ± SD	27.1 ± 4.5	26.4 ± 4.5	26.9 ± 3.2
% TWL after conversion, mean ± SD	36.4 ± 10.8	37.8 ± 12	34.5 ± 5.9
TWL < 20% at last F/U, n (%)	1 (1.8%)	1 (3.8%)	0 (0%)
% EWL after conversion, mean ± SD	90.3 ± 24.1	93.4 ± 23.7	90.5 ± 18.4
Surgical			
Long term complications, n (%)	3 (5.4%)	3 (11.5%)	0 (0%)
Malnutrition/Osteoporosis/Osteopenia, n (%)	1/3 (1.8%)	1/3 (3.8%)	-
Anemia, n (%)	0 (0%)	1/3 (3.8%)	-
Cholecystectomy, liver enzymes disturbances, n (%)	0 (0%)	1/3 (3.8%)	-
Stenosis, n (%)	1/3 (1.8%)	0 (0%)	-
Depression exacerbation, n (%)	1/3 (1.8%)	0 (0%)	-
Conversion/Revisional surgery, n (%)	0 (0%)	0 (0%)	0 (0%)

*BMI* body mass index, *TWL* total weight loss, *EWL* excess weight loss

Long-term complications were reported in 5.4% at 1 year and 11.5% at 3 years, including malnutrition/osteopenia (1.8% and 3.8% at 1 and 3 years), anemia (3.8% at 3 years), biliary/hepatic-related complications (3.8% at 3 years), stenosis (1.8% at 1 year), and depression exacerbation (1.8% at 1 year). No patient required revisional surgery during follow-up after the perioperative period.

At 1-year follow-up after SADI-S conversion, significant improvements were observed in metabolic parameters (Table 4), with a reduction in HbA1c from 5.5 ± 0.7% to 4.6 ± 0.4% (p = 0.02). Among nutritional markers, ferritin increased from 74.7 ± 76.9 to 80 ± 86.8 (p = 0.019), while folic acid levels decreased from 10.1 ± 9.1 to 8.7 ± 5.3 (p = 0.022). No significant changes were found in hemoglobin, iron, albumin, transferrin, vitamin B12, or vitamin D levels. Lipid profiles improved non-significantly, with reductions in total cholesterol, LDL, HDL, and triglycerides. Of note, the proportion of patients receiving vitamin supplementation increased from 40.6% preoperatively to 89.3% postoperatively. Additionally, 23.2% required iron transfusions, and the mean bowel movement frequency was 3.3 ± 1.7 per day.

**Table 4:** Pre and post SADI-S conversion nutritional parameters

Characteristic	before	at 1 year F/U	delta	*p* value
	*n* = 69	*n* = 56 (81.2%)		
HbA1c, mean ± SD	5.5 ± 0.7	4.6 ± 0.4	0.9 ± 0.8	**0.02**
Hb, mean ± SD	13.5 ± 1.7	12.8 ± 1.6	0.7 ± 1.1	0.2
Albumin, mean ± SD	3.9 ± 0.2	4 ± 0.28	(-)0.02 ± 0.3	0.2
Iron, mean ± SD	77.8 ± 26.6	80.2 ± 30.7	(-)2.3 ± 31.7	0.2
Ferritin, mean ± SD	74.7 ± 76.9	80 ± 86.8	(-) 5.2 ± 92.4	**0.019**
Transferrin, mean ± SD	278.7 ± 4 9.8	252.1 ± 62.3	26.5 ± 58.1	0.2
Folic Acid, mean ± SD	10.1 ± 9.1	8.7 ± 5.3	1.3 ± 5.8	**0.022**
B12, mean ± SD	426.4 ± 184.2	469.5 ± 272.6	(-) 43.1 ± 331.3	0.19
Vitamin D, mean ± SD	20.2 ± 12.8	21.6 ± 11.6	(-) 1.4 ± 14.9	0.2
Total Cholesterol, mean ± SD	182 ± 41.2	139 ± 29.5	43 ± 34.2	0.2
LDL, mean ± SD	110.1 ± 32.7	78.5 ± 21.6	31.5 ± 35	0.2
HDL, mean ± SD	51.3 ± 14.8	47.9 ± 9.7	3.3 ± 13.5	0.2
Triglycerides, mean ± SD	120 ± 41.9	78.7 ± 28	41.2 ± 45.3	0.2
Vitamin Supp, n (%)	28 (40.6%)	50 (89.3%)	48%	N/A
Iron transfusion, n (%)	N/A	13 (23.2%)	N/A	N/A
Bowel movements, mean ± SD	N/A	3.3 ± 1.7	N/A	N/A

## Discussion

Conversion procedures for weight-related indications after primary MBS are challenging for both surgeons and patients, with limited evidence guiding the optimal choice and its metabolic and nutritional consequences. This study focuses on conversions to SADI-S following previous MBS for RWG/SCR, with particular emphasis on the metabolic and nutritional impact SADI-S.

Most patients in our cohort were adult females with class III obesity prior to both primary and conversion bariatric surgery, typically having undergone a single previous procedure - most commonly SG. Importantly, the type of prior surgery did not significantly affect response rates (Supplement Table 1). The improvement in glycemic control, along with stable nutritional and metabolic parameters, underscores the potential of SADI-S as an effective conversion option for weight-related indications.

Our findings are consistent with previous studies supporting SADI-S as a viable option for managing weight-related indications after failed MBS. First described by Sánchez-Pernaute et al. in 2015 [14], SADI-S performed as a second stage after SG resulted in an 18-month EWL of 72%, with 88% of patients (*n* = 8) achieving complete diabetes remission. The procedure utilized a 250 cm common channel, and only one patient developed hypoalbuminemia in the first postoperative year, which resolved by year two. Wu et al. [15] reported outcomes for 22 patients who underwent conversion from LAGB to SADI-S for recurrent weight gain, demonstrating a 2-year %EWL of 81% and %TWL of 25%. The longest reported follow-up after primary SADI-S, also by Sánchez-Pernaute et al. [20], included 164 patients with up to 10 years of follow-up, showing a TWL of 34%. Revisional surgery for hypoproteinemia was required in 12 patients (7.3%).

Dijkhorst et al. [21] compared conversions from SG to either SADI (*n* = 66) or RYGB (*n* = 74) for weight-related indications and found significantly greater %TWL at 2 years in the SADI group (26.4% vs. 6.9%). While nutritional deficiencies were observed in both groups, vitamin B12 deficiency was significantly more common following conversion to RYGB.

Zaveri et al. [22] retrospectively reviewed 96 patients who underwent SADI-S, including 64 unplanned and 29 planned two-stage procedures. At a mean follow-up of 24 months, the overall %TWL was 20.5%, with significantly greater weight loss in the planned group compared to the unplanned group (23.1% vs. 19.3%). At 12 months, the authors reported resolution of abnormal levels in HbA1c, fasting glucose, cholesterol, and triglycerides, despite the presence of nutritional deficiencies.

Barajas Gamboa et al. [23] conducted the largest study to date comparing primary SADI-S (*n* = 488) with conversions from SG to SADI-S (*n* = 295), primarily for weight-related indications, with a focus on safety outcomes. They found that while conversion procedures were associated with longer operative times, they did not result in increased morbidity.

Our results further reinforce the role of SADI-S as a safe and effective conversion option for RWG/SCR after various primary MBS procedures. At a mean follow-up of 2.2 years, our cohort demonstrated a mean %TWL of 36.4% at 1 year, 37.8% at 3 years, and 34.5% at 5 years, with corresponding %EWL consistently exceeding 90%—comparable or superior to prior reports. Importantly, only two patients (2.9%) failed to achieve a %TWL > 20%, and none of the eight patients with 5-year follow-up fell below this threshold. These findings support the long-term durability of SADI-S as a revisional strategy for inadequate weight outcomes. It is important to emphasize that these favorable outcomes were consistent across all subgroups, regardless of the type of prior procedure - whether restrictive (LSG, LAGB) or combined restrictive-hypoabsorptive (RYGB, OAGB). Of note, we did not perform subgroup weight-loss analyses within the sleeve cohort comparing re-sleeved and non-re-sleeved patients because of marked group imbalance (7 vs. 38 patients) and probable baseline heterogeneity, including eating behavior, original sleeve characteristics, and demographics. This clinically important issue should be addressed in future studies with larger, more balanced populations.

Metabolic benefits were also notable in our cohort. We observed a significant reduction in HbA1c levels one year postoperatively (from 5.5% to 4.6%, *p* = 0.02), even in a group with relatively low baseline diabetes prevalence. These improvements occurred without significant deterioration in key nutritional markers such as albumin, hemoglobin, or vitamin B12. This emphasizes that conversion to SADI-S achieved durable weight loss and metabolic stability without compromising micronutrient status or inducing anemia, which is a clinically relevant outcome in the setting of conversion surgery.

The marked rise in postoperative vitamin supplementation compliance (from 40.6% to 89.3%) reflects the implementation of a more rigorous and structured follow-up protocol after SADI-S conversion. This proactive approach accounts for the malabsorptive nature of the procedure and highlights improved patient adherence to supplementation recommendations rather than an increase in nutritional deficiencies.

Although folate levels decreased and iron supplementation was required in some patients, the overall complication rate remained acceptable, and long-term revisional surgery after the perioperative period was not required. A modest increase in bowel movement frequency is an expected consequence of the aggressive malabsorptive nature of the SADI-S procedure, which modifies nutrient transit and bile flow to achieve durable metabolic outcomes. Similarly, the need for iron transfusion observed in a subset of patients reflects the malabsorptive physiology of the operation and underscores the importance of vigilant postoperative monitoring. These findings are consistent with those reported by Harker et al. (2025), who noted iron deficiency in 11.4–30.4% of patients at 1 and 3 years after SADI-S, and anemia in up to 26% at these intervals following SADI-S, emphasizing that iron depletion remains a common nutritional concern even under standardized supplementation protocols [24].

The safety profile of SADI-S conversion was also encouraging. No intraoperative complications or perioperative deaths were reported. Postoperative complications occurred in 11.6% of patients within 90 days, with major complications (Clavien-Dindo ≥ 3b) occurring in 5.8% - primarily related to leaks from the re-sleeve staple line. The observed leak rate (7.2%) primarily originated from the re-sleeve staple line rather than the duodeno-ileal anastomosis. This rate aligns with other revisional series (6–10%) [10] and reflects the technical challenges of operating on scarred, less-vascularized tissue after prior stapling. All leaks were promptly managed - three operatively and one conservatively with complete recovery.” All major complications were managed successfully, either operatively or conservatively. These findings are particularly relevant given the complexity and potential risks of revisional MBS.

This study contributes to the limited literature on conversion to SADI-S for weight-related indications, presenting a well-characterized cohort and a standardized surgical approach performed at a high-volume bariatric center. Our inclusion of long-term follow-up data up to 5 years, along with detailed metabolic and nutritional outcomes, provides a comprehensive view of both efficacy and safety. Limitations, however, include the retrospective design and single-center setting, which may limit generalizability. The relatively small number of patients with long-term follow-up, especially at 5 years, may underestimate late complications. In addition, the total small bowel length was not routinely measured or documented intraoperatively. Although the configuration followed the standardized SADI-S technique, minor inter-patient variations may exist and could influence nutritional and bowel function outcomes.

## Conclusion

Conversion to SADI-S is a safe and effective option for patients with RWG/SCR after primary bariatric surgery. It provides durable weight loss, significant metabolic improvement, and acceptable complication rates, with manageable nutritional risks. These findings support its role as a viable revisional strategy for weight-related failure after MBS. Further prospective studies are needed to confirm long-term outcomes and refine patient selection.

### Compliance with Ethical Requirements

## Supplementary Information

Below is the link to the electronic supplementary material.

Supplementary Material 1 (DOCX 14.1 KB)

## Data Availability

No datasets were generated or analysed during the current study.
