# Supplementary material for: SADI-S as a Conversion for Suboptimal Clinical Response and Recurrent Weight Gain Following Bariatric Surgery: A Retrospective Cohort Study
Source: Obes Surg. 2026 Jan 26;36(3):1143–50. doi: 10.1007/s11695-025-08483-3 (PMC13038788; doi:10.1007/s11695-025-08483-3)
Supplement: Supplementary file 1 — Supplementary Material 1 (DOCX 14.1 KB) [file 11695_2025_8483_MOESM1_ESM.docx]

**Supplementary Table 1.**

| **Supp. 1 Comparison between different types of baseline bariatric surgeries** | | | | |
| --- | --- | --- | --- | --- |
| ***p value*** | **Bypass (OAGB + RYGB)** | **LAGB** | **LSG** | **Prior MBS** |
|  | 11 (15.9%) | 13 (18.8%) | 45 (65.2%) | Number of previous MBS, n (%) |
| 0.13 | 41.1 ± 5.7 | 46.7 ± 6.9 | 42.7 ± 6.8 | BMI before conversion, mean ± SD |
| 0.11 | 40.2 (28.0-69.3) | 61.6 (32.8-92.0) | 44.4 (23.8-101.3) | EW before conversion, median (IQR) |
| 0.65 | 40.5 ± 13.7 | 50.1 ± 28.8 | 44.6 ± 19.9 | Delta weight loss, mean ± SD |
| 0.2 | 26.7 ± 5.6 | 30.0 ± 8.7 | 26.4 ± 4.0 | BMI at last F/U, mean ± SD |
| 0.43 | 13.1 (9.8-23.3) | 17.7 (0.8-31.2) | 15.1 (5.7-33.9) | Delta BMI, median (IQR) |
| 0.21 | 5.4 ± 16.6 | 13.1 ± 22.5 | 3.4 ± 10.6 | EW at last F/U, mean ± SD |
| 0.68 | 33.7 (21.7-48.8) | 40.8 (1.6-55.4) | 37.6 (14.6-56.0) | TWL at last F/U, median (IQR) |

*MBS*, metabolic and bariatric surgery; *BMI*, body mass index; *LSG*, Laparoscopic Sleeve Gastrectomy; *LAGB*, Laparoscopic adjustable gastric banding; *OAGB*, One Anastomosis Gastric Bypass; *RYGB*, Roux en Y Gastric Bypass; *BMI*, body mass index; *EW*, Excess Weight; *TWL*, total weight loss
